# Nuclear Receptors as Potential Therapeutic Targets for Myeloid Leukemia

**DOI:** 10.3390/cells9091921

**Published:** 2020-08-19

**Authors:** Pan Pan, Xiao Chen

**Affiliations:** Division of Hematology and Oncology, Department of Medicine, Medical College of Wisconsin, Milwaukee, WI 53226, USA; ppan@mcw.edu

**Keywords:** ATRA, RAR, VDR, PPAR, RXR, AML, CML

## Abstract

The nuclear receptor (NR) superfamily has been studied extensively in many solid tumors and some receptors have been targeted to develop therapies. However, their roles in leukemia are less clear and vary considerably among different types of leukemia. Some NRs participate in mediating the differentiation of myeloid cells, making them attractive therapeutic targets for myeloid leukemia. To date, the success of all-*trans* retinoic acid (ATRA) in treating acute promyelocytic leukemia (APL) remains a classical and unsurpassable example of cancer differentiation therapy. ATRA targets retinoic acid receptor (RAR) and forces differentiation and/or apoptosis of leukemic cells. In addition, ligands/agonists of vitamin D receptor (VDR) and peroxisome proliferator-activated receptor (PPAR) have also been shown to inhibit proliferation, induce differentiation, and promote apoptosis of leukemic cells. Encouragingly, combining different NR agonists or the addition of NR agonists to chemotherapies have shown some synergistic anti-leukemic effects. This review will summarize recent research findings and discuss the therapeutic potential of selected NRs in acute and chronic myeloid leukemia, focusing on RAR, VDR, PPAR, and retinoid X receptor (RXR). We believe that more mechanistic studies in this field will not only shed new lights on the roles of NRs in leukemia, but also further expand the clinical applications of existing therapeutic agents targeting NRs.

## 1. Overview of Nuclear Receptors

The human nuclear receptor (NR) superfamily consists of 48 members that share highly evolutionarily conserved structures. They are composed of several major domains, including the N-terminal regulatory domain with activation function 1 (AF1), the DNA-binding domain, the hinge region, and the ligand-binding domain. The DNA-binding domains consist of two zinc finger motifs that are responsible for the binding specificity of their responsive elements, and the ligand-binding domains might have an additional AF2 region that is regulated by co-regulator interaction. Most of the NRs are activated by endogenous small lipophilic ligands, including steroid hormones, thyroid hormones, lipophilic vitamins, and cholesterol metabolites. However, some of the NRs are considered as orphan receptors since their endogenous physiological ligands have not yet been identified [1,2,3].

Ligand binding induces conformational changes within the NRs and triggers their translocation into the nucleus, where they can recognize and bind to their responsive elements within the regulatory regions of target genes to regulate gene transcription [2]. NRs can function as either monomers or homodimers/heterodimers, though dimerization is a general mechanism to increase ligand binding affinity, response specificity, and diversity. Steroid hormone receptors usually form homodimers [4], while other receptors, such as retinoic acid receptor (RAR), vitamin D receptor (VDR), peroxisome proliferator-activated receptor (PPAR), and retinoid X receptor (RXR) can form both homodimers and heterodimers [5]. However, RXR serves as a preferable heterodimerization partner for RAR, VDR, PPAR, and some orphan receptors [6]. 

Nuclear receptor responsive elements share a conservative sequence core AGGTCA motif with modifications and duplications such as direct, inverted, or everted repeats [5]. These modifications can be recognized selectively by different NRs [1,2,7]. Retinoic acid (RA) induces pleiotropic effects on cellular proliferation and differentiation through binding to members of the RAR subfamily (RAR-α, RAR-β, and RAR-γ) [8]. Upon RA binding, RAR forms heterodimers with RXR and regulates downstream target genes through retinoic acid responsive elements (RAREs), which are characterized by two direct repeats of the sequence core motifs separated by nucleotides with various sizes [8]. VDR is activated by the active form of vitamin D. The heterodimers of VDR/RXR bind to vitamin D responsive elements (VDREs), which contain direct or everted repeats of the sequence core motifs separated by three or six nucleotides [9]. The PPAR subfamily members, such as PPAR-α, PPAR-β/δ, and PPAR-γ, are fatty acid-activated transcription factors [10]. Upon ligand binding, heterodimers of PPAR/RXR bind to peroxisome proliferator responsive elements (PPREs), which contain the sequence core motifs directionally aligned with a single nucleotide spacer [11]. PPARs regulate a variety of downstream target genes that are involved in development, differentiation, and metabolism. 

Ligand binding can further influence the interaction of NRs with co-regulators, either coactivators or corepressors, by changing the conformation of the AF2 region. Co-regulators function as members of large complexes, such as nuclear receptor corepressor (NCoR) and silencing mediator for retinoid and thyroid hormone receptors (SMRT). These complexes regulate the transcription of target genes through interacting with other transcription factors and changing the chromatin landscape. The interactions between co-regulators and the chromosome are dependent on cell/tissue types, which further enhance the specificity of NRs [12,13]. 

The downstream target genes of NRs control a wide variety of biological processes, not only in normal physiologic processes such as embryonic development, reproduction, metabolism, and cell proliferation and differentiation, but also in pathological conditions including cancer, inflammation and metabolic disorders [13,14,15,16,17]. Evidently, NRs have been the therapeutic targets for many diseases, accounting for 10–15% of United States Food and Drug Administration (FDA)-approved therapeutic agents [18], especially for solid tumors such as breast cancer [19], lung cancer [20], and prostate cancer [21]. 

## 2. Potential Roles of NRs in Acute Myeloid Leukemia

There have been estimated 19,940 new cases and 11,180 new deaths of acute myeloid leukemia (AML) in the US in 2020, making it the most deadly type of leukemia [22]. AML is a heterogeneous blood cancer, and its subtypes are characterized by a hematopoietic differentiation arrest at various stages. According to the French-American-British (FAB) classification system, AML can be morphologically divided into eight subtypes (M0–M7). Strikingly, more than 700 chromosomal aberrations have been identified in leukemic cells of AML patients [23]. 

Among all subtypes of AML, acute promyelocytic leukemia (APL, M3) is unique with regard to clinical manifestation, cytogenetic abnormality, and response to differentiation therapy. APL is relatively rare with an estimated incidence of 0.1 per 100,000 in the Western countries and accounts for 5–10% of all AML cases [24]. The molecular hallmark of APL is the presence of a balanced reciprocal translocation between chromosomes 15 and 17 [t(15;17) (q22;q21)], resulting in the *promyelocytic leukemia (PML)-RAR-α* fusion gene. More than 98% of APL patients carry the *PML-RAR-α* fusion gene [8,25]. 

In normal cells, RAR forms heterodimers with RXR. In the absence of RA, the RAR/RXR heterodimers recruit corepressor complexes including NCoR and SMRT, as well as negative regulators of the chromatin such as histone deacetylase (HDAC), histone methyltransferase (HMT), or DNA methyltransferase (DNMT). These corepressor complexes lead to histone deacetylation, chromatin condensation, and transcription repression. Upon RA activation at physiological levels (about 0.01 μM), ligand binding induces conformational change in the RAR/RXR heterodimers which trigger the release of corepressor complexes and the recruitment of coactivator complexes with histone acetyl transferase (HAT)- or ATP-dependent nucleosome remodeling activities. These coactivator complexes trigger histone acetylation, chromatin de-condensation, and transcription activation [26].

In APL cells, the PML-RAR-α fusion protein can form heterodimers with RXR. More importantly, the fusion protein markedly enhances the binding strength between RXR and NCoR, thereby constantly suppressing gene transcription and the RA signaling pathway [27]. One of the downstream target genes is the transcriptional factor PU.1 that is crucial to induce normal hematopoietic differentiation [27,28]. Therefore, the PML-RAR-α fusion protein acts in a dominant negative manner and blocks hematopoietic cell differentiation, leading to an arrest at the early stage as promyelocytic cells [8,25,26,29]. In addition, this fusion protein also forms a heterodimer with PML and acts as a dominant negative inhibitor against the normal functions of PML, which regulate cellular growth arrest and apoptosis [30,31]. 

Conventional chemotherapy targets rapid-dividing cancer cells, but also kills normal cells, such as bone marrow stem and progenitor cells and gut epithelial cells, making it highly toxic to the patients. In contrast, induction therapy or differentiation therapy, first proposed by Leo Sachs [32], aimed to re-activate signaling pathways that are bypassed or suppressed during tumorigenesis. These therapies force cancer cells to resume regular cell cycles and to differentiate, thereby inducing terminal differentiation and/or apoptosis to reverse the malignant phenotype [33,34]. In the late 1980s, ATRA was introduced and quickly became the first game changer for APL [35,36]. At a pharmacologic dosage (0.1–1 μM), ATRA is able to dissociate NCoR complex from the PML-RAR-α fusion protein and recruit the nuclear coactivator to induce differentiation and apoptosis of promyelocytes [26,29]. The transcriptional expression of PU.1 is restored upon ATRA treatment [28]. ATRA also causes degradation of the PML-RAR-α fusion protein through proteasome activation and ubiquitination [29,37,38]. RXR is then released from the fusion protein and is able to form RAR/RXR heterodimers, leading to the restoration of RA signaling and cell differentiation. Moreover, ATRA treatment also induces the relocation of PML in the nuclear bodies and the recovery of PML functions [30,31]. Early clinical studies using ATRA as a single agent demonstrated promising results. For example, a Shanghai group first observed a 85% complete remission (CR) rate in 1988 [35]. The first North American Intergroup study found a 72% CR rate, which was equivalent to the rates obtained by the conventional treatment using cytarabine and daunorubicin [39]. To date, the success of ATRA in treating APL still represents the best example of differentiation therapy in cancer [40]. Later, the introduction of arsenic trioxide (ATO) became the second game changer for APL. ATO has been shown to bind to the PML-RAR-α fusion protein and to cause its degradation through SUMOylation, ubiquitination, and proteasome-dependent mechanisms. As a result, ATO can induce apoptosis and promote the differentiation of APL cells [41,42]. When ATO was given to APL patients as a single agent, high CR rates with relatively long-term remissions were observed in two Chinese clinical studies [43,44]. More importantly, many clinical studies demonstrated statistically significant improvement in clinic outcomes in patients who received the ATRA-ATO combinational regimen compared to patients on ATRA-chemotherapy [45,46,47,48,49]. With the combination of ATRA and ATO, chemotherapy may be omitted in low-risk patients. Differentiation syndrome has been observed in APL patients that were treated with ATRA-ATO, which could be managed by steroid treatment or the discontinuation of the therapy [50].

Combining ATRA with ligands of other NRs, especially PPAR-γ [51,52,53,54], have been developed with the hope of further enhancing differentiation therapy. For example, using ATRA-sensitive NB4 cells and ATRA-resistant NB4-derived subline MR2 cells, Tabe et al. showed that PPAR-γ ligand 2-cyano-3,12-dioxooleana-1,9-dien-28-oic acid (CDDO) enhanced pro-apoptotic and differentiating effects of ATRA in NB4 cells and partially reversed ATRA resistance in MR2 cells. The combination of ATRA and CDDO synergistically induced the expression of RAR-β2 partially through enhancing H3-Lys9 acetylation on the promotor region of *RAR-β2* gene, thereby increasing the binding between RAR and RAREs in both ATRA-sensitive and -resistant APL cells [55]. These results suggest that inhibiting HDAC might sensitize APL cells to ATRA, which would be critical for patients who carry other variants of 17q chromosome translocation, such as promyelocytic leukemia zinc finger protein PLZF-RAR-α [t(11;17)], or patients who relapse. Indeed, several studies have shown encouraging results, where combining HDAC inhibitors with ATRA reversed the transcriptional repression using patients’ primary cells and mouse models [56,57,58].

In contrast to APL patients who harbor a predominant driver mutation, patients with non-APL subtypes of AML are more heterogenous and may carry various molecular mutants. Intensive induction chemotherapy and post-remission consolidation therapies have been widely used for treating these patients. New strategies have also been developed with the hope of prolonging survival and decreasing the risk of relapse [59,60]. For example, the incorporation of a humanized anti-CD33 monoclonal antibody conjugated with calicheamicin (gemtuzumab ozogamicin) into induction therapy has demonstrated a reduced risk of relapse and improved survival in patients with intermediate- to favorable-risk in randomized trials [61,62,63,64,65,66]. In addition, vaccines or autologous dendritic cells fused with patient-derived AML cells are designed to stimulate patients’ own T cells to recognize AML-specific epitopes. Other strategies include tyrosine kinases inhibitors (TKIs), hypomethylating agents, FLT3 inhibitors, and isocitrate dehydrogenase inhibitors [60].

Given the robust differentiating effects of ATRA on APL cells, some studies have tried to target RAR in non-APL subtypes of AMLs [54]. For example, treating HL-60 cells, a widely used AML cell line, with ATRA has been shown to induce their apoptosis/differentiation [54]. However, in one clinical trial, additional ATRA treatment has not shown clinical benefits in terms of overall survival (OS) or disease-free survival (DFS) in patients who were treated with chemotherapies [67]. On the other hand, attempts have been made to target other NRs or to combine different NR ligands to improve the efficacy of differentiation therapy. PPAR-γ ligands alone have been shown to induce differentiation, promote apoptosis, and suppress proliferation of multiple AML cell lines (HL-60, KG-1, Mono-MAC6, and THP-1) [53,68], as well as primary cells from AML patients [54,69,70,71,72]. Multiple anti-leukemic effects have been mediated by PPAR-γ ligands, such as activating c-Jun NH2-terminal kinase (JNK) and p38 mitogen-activated protein kinase (p38 MAPK), inhibiting extracellular-signal-regulated kinase (ERK), the production of reactive oxygen species, and inducing cell cycle arrest [68]. However, in a small clinical study, five patients with refractory/relapsed AML received CDDO for five days, and no significant clinical benefits have been observed, except one patient showed decreased bone marrow blasts and monocytes [72]. Furthermore, Konopleva et al. demonstrated that combining PPAR-γ ligand CDDO, RXR agonist LG100268 with ATRA induced a stronger differentiation effect compared to that of ATRA alone in HL-60 cells [54]. Similar results were observed in HL-60 cells and primary cells from AML patients when combining ATRA with RXR agonists, which were mediated by the induction of PU.1 [73,74]. Different results have been reported when using different NR agonists and AML cell lines (See Supplementary Materials). Konopleva et al. demonstrated that CDDO and LG100268 induced apoptosis and differentiation of HL-60 cells [69]. On the other hand, Sanchez et al. observed that RXR agonist bexarotene in combination with liver X receptor (LXR) agonists (T0901317 or GW3965), but not PPAR-γ ligand rosiglitazone, induced potent differentiation and cytotoxicity in THP-1 cells and primary cells from AML patients [74].

In addition, as the preferable heterodimer partner, RXR ligand bexarotene has been reported to be safe in patients and showed some anti-leukemic activities in a small phase I clinical trial enrolling 27 AML patients [75]. Further studies, especially clinical trials, are warranted to evaluate the efficacy of adding RXR ligands to the standard chemotherapies in non-APL AML patients.

Similar to ATRA, vitamin D also plays a regulatory role in the hematopoiesis, thus it has been suggested as a differentiating agent in the treatment of AML [76]. 1,25-dihydroxyvitamin D3 has been shown to induce cell differentiation through regulating multiple signaling pathways, such as ERK, JNK, phosphoinositide 3-kinase (PI3K), protein kinase B (PKB), and protein kinase C (PKC), as well as cyclin-dependent kinase (CDK) inhibitors (CKIs) such as p21 and p27 [76,77,78,79]. As more people have been working indoors, vitamin D insufficiency/deficiency has become a global health issue [80]. Epidemiologic and observational studies have demonstrated an inverse relationship between circulating 25-hydroxvitamin D and most types of cancer including leukemia [9,81,82,83,84,85]. The combination of calcitriol, a synthetic form of vitamin D3, with azacytidine, the main demethylating agent for the treatment of myelodysplastic syndrome, showed synergistic inhibition on the proliferation of HL-60 and MOLM13 cells [82]. In addition, combining 1,25-dihydroxyvitamin D3 or its analogs with dimethyl fumarate, a nuclear factor erythroid-derived 2-like 2 (NFE2L2 or Nrf2) activator and clinically approved agent for multiple sclerosis and psoriasis, induced strong synergistic pro-differentiating effects in HL-60 cells through cooperatively upregulating VDR and Nrf2 [86]. Another study combined calcitriol with glycogen synthase kinase 3 (GSK3) inhibitors and showed that HL-60 and OCI-AML3 cells were able to undergo further differentiation upon treatment. They also demonstrated that GSK3 inhibition sensitized primary cells from AML patients to calcitriol-induced differentiation through an increased cell cycle arrest in G0-G1 phase and a decreased expression of cyclin A [87]. In addition, the combination of calcitriol and GSK3 inhibitor induced phosphorylation at Ser208 of VDR, resulting in enhanced VDR transcriptional activities and the activation of JNK pathway [87]. These results suggest that vitamin D or its analogs can potentially be used as sensitizing agents in combination with other therapies to improve treatment outcomes for AML patients. 

In summary, other than the breakthrough of ATRA therapy for APL, there has been limited success in developing NR-based therapies in other subtypes of AML. There are several limitations in the research of this area. First, most of the research conducted so far used *in vitro* cell culture systems, and only a few studies addressed the complex roles of NRs *in vivo* in the setting of leukemia. Second, various cell lines are used. This heterogeneity may partially explain the mixed results from different studies. In particular, differences in expression levels of various NRs should be taken into consideration when comparing studies using different cell lines. Third, in studies using primary cells of AML patients, only a few studies [69,71,72] listed the disease stages of patients. The expression levels of NRs on these patient-derived samples were also not fully evaluated. Future research needs to focus on the genomic evaluation of NRs in the pathophysiology of non-APL subtypes of AML. Better understanding of distribution, expression, function, and crosstalk of NRs may provide more insights into developing NR-based therapies for these patients.

## 3. Potential Roles of NRs in Chronic Myelogenous Leukemia

There have been estimated 8,450 new cases and 1,130 new deaths of chronic myelogenous leukemia (CML) in the US in 2020 [22]. The driver mutation to cause CML results from a reciprocal translocation between chromosomes 9 and 22 [(t(9;22) (q34;q11.2)], combining the *abelson murine leukemia (ABL) proto-oncogene* on chromosome 9 with the *breakpoint cluster region protein (BCR)* sequences on chromosome 22 to form the *BCR-ABL* fusion gene [88,89]. All different variants of BCR-ABL fusion proteins contain the common ABL domains, but there are three breakpoint cluster regions for BCR protein: major (M-BCR), minor (m-BCR), and micro (µ-BCR), which correspond to three fusion proteins P210, P190, and P230, respectively [88,89]. Fusion protein P210 has been found in more than 98% of all CML patients [88]. Multiple signaling proteins can interact with the BCR-ABL fusion proteins through various regulatory domains and become phosphorylated, which in turn activate a wide range of signaling pathways, including Ras, PI3K, PKB, JNK, Src kinases, and their respective downstream targets [90]. 

The use of TKIs has revolutionized the treatment of CML. Imatinib has achieved more than 80% of complete cytogenetic responses and a long-term OS rate higher than 90%. In addition, several imatinib discontinuation trials have demonstrated that 40% of patients remained in stable remission after discontinuing treatment, and newer generations of TKIs further improved the outcomes [91]. Therefore, CML now has a relatively low mortality rate among all types of leukemia [92]. Though TKIs are considered well-tolerated, it has been known that they cause adverse effects, including gastrointestinal, cardiovascular, dermatologic, and hepatic toxicities in subgroups of CML patients. These long-term toxicities can be managed and even abrogated by the discontinuation of the TKI treatment if the disease progression is not a concern [93]. In addition, patients may become resistant to TKI treatment. The BCR-ABL fusion proteins can initiate and activate other non-receptor tyrosine kinases and their downstream signaling pathways, which can promote TKI resistance [94]. Furthermore, genomic instability and additional genetic mutations of the *BCR-ABL* fusion gene, as well as drug efflux, may also cause TKI resistance [94].

Encouraging results have also been reported both *in vitro* [95,96] and *in vivo* [97] by combining PPAR-γ ligands and TKIs. Using a CML cell line (K-562) and leukemia stem cells (LSCs) from CML patients, Prost et al. demonstrated that adding PPAR-γ agonist pioglitazone to TKIs significantly decreased the clonogenic potential of CML cells through downregulating the expression of *signal transducer and activator of transcription 5* (*STAT5*) and its target genes [95]. Similarly, another group showed synergistic anti-leukemic efficacy in K-562 cells when combining synthetic PPAR-γ agonists with TKIs [96]. In addition, Prost et al. gave pioglitazone temporarily ranging from six months to 32 months to three CML patients who were on the treatment of imatinib. All three patients achieved sustained complete molecular remission, up to 4.7 years after the discontinuation of pioglitazone [95]. Based on the promising results from this pilot study, a clinical study enrolled 24 CML patients and co-administered pioglitazone in addition to imatinib [97]. The cumulative incidence of molecular response 4.5 significantly increased in patients with the combinational therapies compared to those received imatinib alone. These results suggest a strong potential of using PPAR-γ agonists as sensitizing agents in combination with TKIs, especially through their effects on LSCs [95,98,99,100,101,102]. Currently, the combination of pioglitazone and imatinib is under investigation in a prospective randomized trial (NCT02767063). 

Similar results have also been observed with PPAR-α, another member within the PPAR subfamily. PPAR-α agonists significantly enhanced the anti-leukemic effects of imatinib in KCL22 cells and CD34^+^ primary cells through upregulating *human organic cation transporter* (*hOCT1*) gene expression and increasing the uptake of imatinib by CML cells [103]. Based on the predicting feature of *hOCT1* gene expression in terms of therapeutic response to imatinib in CML patients [104,105,106], the same group investigated the efficacy of upregulating *hOCT1* by other NRs [107]. Among 13 NR pathways tested, agonists of pregnane X receptor (PXR, rifampicin, SR12813, and T-0907317), RAR (ATRA), and RXR (9-*cis*-retinoic acid) were able to increase the expression of *hOCT1* in KCL22 cells and/or peripheral blood mononuclear cells from CML patients [107], suggesting that PXR, RAR, and RXR signaling pathways can be novel combinational partners for TKIs in therapeutic regimens.

In summary, CML patients predominantly carry the *BCR-ABL* translocation and TKIs have become the backbone of therapeutic strategies for this disease. Intriguingly, given the availability of existing NR agonists in the market and the observation that some NR agonists can upregulate the expression of *hOCT1*, an independent predictor of therapeutic response, combining NR agonists and TKIs could shed new lights on the development of novel therapeutic regimens for CML patients.

## 4. Conclusions and Perspectives

NRs play various roles in biologic processes and regulate even more diverse downstream signaling networks. ATRA, vitamin D and its analogs, as well as PPAR agonists have been studied and reported to inhibit proliferation, induce differentiation and apoptosis in various leukemic cells. Combinations of these agents have also been tested and shown synergistic effects, suggesting that multiple NRs are involved in the pathophysiology of leukemia. In addition, many NR agonists, such as vitamin D and PPAR agonists, can function as sensitizing agents for current therapies. This has the potential of reducing the dosage of chemotherapy, thereby lowering the treatment-associated toxicity and improving clinical outcomes. This can be a new and attractive direction for targeting NRs in leukemia, especially on top of the success of TKI regimens in CML. We listed examples of studies on NR agonists-based treatments for AML and CML in Table 1. There are still many outstanding questions that need to be addressed before there is a hope of reproducing the success of ATRA therapy for APL in other types of leukemia. For example, what are the mechanisms responsible for the aberrant expression of some NRs in leukemic cells? How does the leukemic microenvironment contribute to altering the expression of those NRs? How can we enhance sensitivity of leukemic cells to various differentiating reagents? What can NR-targeted therapies contribute in an era of immunotherapy of leukemia, such as chimeric antigen receptor-T cell (CAR-T) therapy? Is there a potential involvement of patients’ immune system in NR-based therapies? How to target non-canonical NR signaling to improve leukemia therapy? Therefore, comprehensive genomic studies of the NR network in leukemogenesis are much needed. This line of research may provide more integrated insights into the roles of NRs in leukemia, including their expression levels, distribution, functional interactions, and mechanisms of action. More investigations also need to focus on exploring the complex interactions between NR-mediated regulation on the chromatin landscape and disease-driven mutants. In addition, comprehensive genomic analysis could provide NR network profiles for individual patients, thus identifying which patient is likely to respond to a particular NR-based therapy. This will substantially enhance the potential applications of precision medicine. Finally, given the availability of numerous approved NR agonists and antagonists, we could expect that more research in this area will lead to repurposing of these drugs for treating leukemia patients. 

## Figures and Tables

**Table 1:** Examples of studies on selected NR agonists-based treatments in AML and CML.

Types of Leukemia	Subtypes	Cytogenetic Abnormalities	NR-Agonists Based Treatments	Cells/Patients	Effects	Ref.
AML	APL	*PML-RAR-α, PLZF-RAR-α*	ATRA	24 APL patients	Induced complete remission without bone marrow hypoplasia	[35]
			ATRA	346 APL patients	Improved disease-free and overall survival as compared with chemotherapy alone	[39]
			ATRA + ATO	61 APL patients	Improved complete remission rates and disease-free survival	[45]
			ATRA + ATO + chemotherapy	124 APL patients	Improved relapse-free and failure-free survival	[46,48]
			ATRA + ATO	156 APL patients	Induced complete remission and improved 2-year event-free survival rates and overall survival	[47]
			ATRA + ATO	276 APL patients	Induced complete remission and improved the event-free survival, cumulative incidence of relapse, and overall survival at 50 months	[49]
			PPAR-γ agonist + ATRA	ATRA-sensitive NB4 cells	Sensitized ATRA-induced effects, promoted apoptosis, and enhanced differentiation of NB4 cells	[55]
				ATRA-resistant NB4-derived subline MR2 cells	Partially reversed ATRA resistance in MR2 cells	
	Non-APL subtypes	Heterogeneous	ATRA	HL-60 cells	Induced apoptosis and differentiation	[54]
			ATRA + chemotherapy	Patients with non-APL subtypes of AML	No significant clinical benefit in terms of overall survival or disease-free survival	[67]
			PPAR-γ agonist	HL-60, KG-1, Mono-MAC6, and THP-1 cells	Induced differentiation, promoted apoptosis, and suppressed proliferation, activated JNK and p38 MAPK pathways, inhibited ERK pathway, enhanced the production of reative oxygen species, and induced cell cycle arrest	[53,68]
				Primary cells from AML patients		[54,69,70,71,72]
			PPAR-γ agonist (ODDO)	5 patients with refractory/relapsed AML	No significant clinical benefit, except one patient showed decreased bone marrow blasts and monocytes	[72]
			PPAR-γ agonist + RXR agonist + ATRA	HL-60 cells	Synergistic anti-leukemic effects, promoted apoptosis and induced differentiation, suppressed ERK pathway, decreased the expression of anti-apoptotic Bcl-2, and increased the expression of pro-apoptotic Bax	[54]
			RXR agonist + LXR agonist	THP-1 cells	Induced differentiation and enhanced cytotoxicity	[73,74]
				Primary cells from AML patients		
			RXR agonist (bexarotene)	27 AML patients who either had refractory/relapsed diseases, or were not eligible for standard cytotoxic chemotherapy	Four (15%) patients showed reduced bone marrow blasts to less than 5%; 11 (41%) patients had improved platelet counts, 7 (26%) patients had improved neutrophil counts; and 3 patients with relapsed AML survived more than one year.	[75]
			VDR agonist + azacytidine (demethylating agent)	HL-60 and MOLM13 cells	Synergistic inhibition on leukemic cell proliferation	[82]
			VDR agonist + dimethyl fumarate (Nrf2 activator)	HL-60 cells	Synergistic pro-differentiating effects through cooperatively upregulating VDR and Nrf2	[86]
			VDR agonist + GSK3 inhibitor	HL-60 and OCI-AML3 cells	Synergistic pro-differentiating effects through an increased cell cycle arrest in G0-G1 phase and a decreased expression of cyclin A, induced phosphorylation at Ser208 of VDR, enhanced VDR transcriptional activities, and activated JNK pathway	[87]
CML		*BCR-ABL*	PPAR-γ agonist +TKIs	K562	Decreased the clonogenic potential of CML cells through downregulating the expression of *STAT5* and its target genes *HIF2α* and *Cbp/p300-interacting transactivator 2*	[95]
				Leukemia stem cell from CML patients		
			PPAR-γ agonist (pioglitazone) + imatinib	3 CML patients	Achieved sustained complete molecular remission, up to 4.7 years after the discontinuation of pioglitazone	[95]
			PPAR-γ agonist (pioglitazone) + imatinib	24 CML patients	The cumulative incidence of molecular response 4.5 was 56% by 12 months, in comparison with 23% in the parallel control group with patients that only received imatinib	[97]
			PPAR-α agonist + imatinib	KCL22 cells	Synergistic anti-leukemic effects through upregulating *hOCT1* gene expression and increasing the uptake of imatinib by CML cells	[103]
				Leukemia stem cell from CML patients		

## Supplementary Materials

The following are available online at https://www.mdpi.com/2073-4409/9/9/1921/s1, Table S1. Examples of studies on selected NR agonists-based treatments in AML and CML.
