# Supplementary material for: Nuclear Receptors as Potential Therapeutic Targets for Myeloid Leukemia
Source: Cells. 2020 Aug 19;9(9):1921. doi: 10.3390/cells9091921 (PMC7563802; doi:10.3390/cells9091921)
Supplement: Supplementary file 1 [file cells-09-01921-s001.pdf]

Table 1. Examples of studies on selected NR agonists-based treatments in AML and CML

Types of leukemia	Subtypes	Cytogenetic abnormalities	NR-agonists based treatments
AML	APL	PML-RAR-α, PLZF-RAR-α	ATRA
			ATRA
			ATRA + ATO
			ATRA + ATO + chemotherapy
			ATRA + ATO
			ATRA + ATO
			PPAR-γ agonist + ATRA
	Non-APL subtypes	Heterogeneous	ATRA
			ATRA + chemotherapy
			PPAR-γ agonist
			PPAR-γ agonist (ODDO)
			PPAR-γ agonist + RXR agonist + ATRA
			RXR agonist + LXR agonist
			RXR agonist (bexarotene)
			VDR agonist + azacytidine (demethylating agent)
			VDR agonist + dimethyl fumarate (Nrf2 activator)
			VDR agonist + GSK3 inhibitor
CML		BCR-ABL	PPAR-γ agonist +TKIs
			PPAR-γ agonist (pioglitazone) + imatinib
			PPAR-γ agonist (pioglitazone) + imatinib
			PPAR-α agonist + imatinib

**Cells/Patients**
24 APL patients
346 APL patients
61 APL patients
124 APL patients
156 APL patients
276 APL patients
ATRA-sensitive NB4 cells
ATRA-resistant NB4-derived subline MR2 cells
HL-60 cells
Patients with non-APL subtypes of AML
HL-60, KG-1, Mono-MAC6, and THP-1 cells
Primary cells from AML patients
5 patients with refractory/relapsed AML
HL-60 cells
THP-1 cells
Primary cells from AML patients
27 AML patients who either had refractory/relapsed diseases, or were not eligible for standard cytotoxic chemotherapy
HL-60 and MOLM13 cells
HL-60 cells
HL-60 and OCI-AML3 cells
K562
Leukemia stem cell from CML patients
3 CML patients
24 CML patients
KCL22 cells
Leukemia stem cell from CML patients

Effects
Induced complete remission without bone marrow hypoplasia
Improved disease-free and overall survival as compared with chemotherapy alone
Improved complete remission rates and disease-free survival
Improved relapse-free and failure-free survival
Induced complete remission and improved 2-year event-free survival rates and overall survival
Induced complete remission and improved the event-free survival, cumulative incidence of relapse, and overall survival at 50 months
Sensitized ATRA-induced effects, promoted apoptosis, and enhanced differentiation of NB4 cells
Partially reversed ATRA resistance in MR2 cells
Induced apoptosis and differentiation
No significant clinical benefit in terms of overall survival or disease-free survival
Induced differentiation, promoted apoptosis, and suppressed proliferation, activated JNK and p38 MAPK pathways, inhibited ERK pathway, enhanced the production of reative oxygen species, and induced cell cycle arrest
No significant clinical benefit, except one patient showed decreased bone marrow blasts and monocytes
Synergistic anti-leukemic effects, promoted apoptosis and induced differentiation, suppressed ERK pathway, decreased the expression of anti-apoptotic Bcl-2, and increased the expression of pro-apoptotic Bax
Induced differentiation and enhanced cytotoxicity
Four (15%) patients showed reduced bone marrow blasts to less than 5%; 11 (41%) patients had improved platelet counts, 7 (26%) patients had improved neutrophil counts; and 3 patients with relapsed AML survived more than one year.
Synergistic inhibition on leukemic cell proliferation
Synergistic pro-differentiating effects through cooperatively upregulating VDR and Nrf2
Synergistic pro-differentiating effects through an increased cell cycle arrest in G0-G1 phase and a decreased expression of cyclin A, induced phosphorylation at Ser208 of VDR, enhanced VDR transcriptional activities, and activated JNK pathway
Decreased the clonogenic potential of CML cells through downregulating the expression of STAT5 and its target genes HIF2α and Cbp/p300-interacting transactivator 2
Achieved sustained complete molecular remission, up to 4.7 years after the discontinuation of pioglitazone
The cumulative incidence of molecular response 4.5 was [illegible] by 12 months, in comparison with [illegible] in the parallel control group with patients that only received imatinib
Synergistic anti-leukemic effects through upregulating hOCT1 gene expression and increasing the uptake of imatinib by CML cells

**Ref.**
35
39
45
46, 48
47
49
55
54
67
53, 68
54, 69-72
72
54
73, 74
75
82
86
87
95
95
97
103
